# Effect of different single and combined antihypertensive drug regimens on the mortality of critical care patients

**DOI:** 10.3389/fphar.2024.1385397

**Published:** 2024-08-28

**Authors:** Yipeng Fang, Xianxi Huang, Junyu Shi, Chunhong Ren, Xin Zhang

**Affiliations:** ^1^ Laboratory of Molecular Cardiology, The First Affiliated Hospital of Shantou University Medical College, Shantou, Guangdong Province, China; ^2^ Laboratory of Medical Molecular Imaging, The First Affiliated Hospital of Shantou University Medical College, Shantou, Guangdong Province, China; ^3^ Shantou University Medical College, Shantou, Guangdong Province, China; ^4^ Department of Cardiology, The First Affiliated Hospital of Shantou University Medical College, Shantou, Guangdong Province, China; ^5^ International Medical Service Center, The First Affiliated hospital of Shantou University Medical College, Shantou, Guangdong Province, China

**Keywords:** antihypertensives, prognosis, intensive care unit, hypertension, angiotensin converting enzyme inhibitors, angiotensin-receptor blockers, previously exposure

## Abstract

**Objective:**

To investigate the effect of different single and combined pre-admission antihypertensive drug regimens on the prognosis of critically ill patients.

**Methods:**

We performed a retrospective cohort study using data from the Medical Information Mart for Intensive Care-IV (MIMIC-IV) database. All initial ICU admission records of patients with hypertension and previous antihypertensive exposure before ICU admission were included. Our primary outcome was 90-day mortality. Propensity score matching (PSM) and inverse probability of treatment weighting (IPTW) were used to balance the distribution of baseline characteristics. Logistic regression analysis and subgroup analysis were performed to determine the independent effect of different single and combined antihypertensive drug regimens on 90-day mortality.

**Results:**

A total of 13,142 patients were included in the final analysis. The 90-day mortality rate in the combined groups is lower than that in the single therapy group (10.94% vs 11.12%), but no statistical significance was found in the original cohort (*p* = 0.742). After adjustment for potential confounders, the significantly decreased 90-day mortality rate was found in the combined groups (10.78% vs 12.65%, *p* = 0.004 in PSM; 10.34% vs 11.90%, *p* = 0.007). Patients who were exposed to either ACEIs or ARBs had a better prognosis than those not exposed (7.19% vs 17.08%, *p* < 0.001 in single antihypertensive groups; 8.14% vs18.91%, *p* < 0.001 in combined antihypertensive groups). The results keep robustness in the PSM and IPTW cohorts. In the logistic regression model analysis, combined therapy was associated with a 12%–20% reduced risk of 90-day death after adjusting potential confounders (OR 0.80–0.88, all *p* < 0.05), while exposure to ACEIs or ARBs was associated with the decreased risk of 90-day death by 52%–62% (OR 0.38–0.48, all *p* < 0.001) and 40%–62% (OR 0.38–0.60, all *p* < 0.001) in the single and combined therapy groups, respectively. The results were still robust to subgroup analysis.

**Conclusions:**

Pre-admission combined antihypertensive therapy is associated with a significantly lower risk of death than exposure to single antihypertensives in critically ill patients. Meanwhile, either ACEIs or ARBs seem to be the optimal candidates for both single and combined therapy. Further high-quality trials are needed to confirm our findings.

## 1 Introduction

Hypertension is a well-known risk factor for several cardiovascular and cerebrovascular events and also increases the risk of renal failure, disability and mortality (Mills et al., 2016). Global adult prevalence of hypertension is 31.1%, with 28.5% in high-income countries and 31.5% in middle- and low-income countries (Mills et al., 2016). Long term effective blood pressure control is essential to reduce the risk of stroke, heart attack, heart failure and to improve the outcomes in patients with hypertension (Emrich et al., 2019). There is a wide range of classical antihypertensive drugs available in clinic, which lower blood pressure through different mechanisms and therefore exert different additional effects. For example, the long-term use of angiotensin converting enzyme inhibitors (ACEIs)/angiotensin receptor blockers (ARBs) can significantly improve the expression of ACE2, which is one of the crucial regulators of the renin–angiotensin–aldosterone system (RAAS) and help to inhibit systemic inflammatory reaction (Kriszta et al., 2021). ACEIs and ARBs have also been reported to participate in maintaining epithelial and endothelial barrier functions (Salgado et al., 2010). The exposure of β-blockers can effectively improve coronary perfusion, increase stroke volume, reduce myocardial oxygen consumption and inhibit the loss of cardiac myocytes by blocking the direct cardio-toxic effects of catecholamine (Macchia et al., 2012). Calcium channel blockers (CCBs) can effectively reduce oxidative stress and the secretion of inflammatory cytokines (Li et al., 2009). Due to the abundant but heterogeneous pharmacological effects of different antihypertensive agents, the optimal treatment options of antihypertensive agents have always received wide attention (Jiang et al., 2009; Christiansen et al., 2020; Gao et al., 2022).

Previous studies indicated that outpatient and pre-hospital antihypertensive drug exposure can influence the clinical prognosis for different populations. Jeffery et al. reported that outpatient use of ARBs was related to the improved prognosis compared with other kinds of antihypertensive drugs in the acute viral respiratory illness (AVRI) (Jeffery et al., 2021). They also found that outpatient exposure of ACEIs were associated with the decreased risk of death for patients with AVRI (Jeffery et al., 2021). However, during the COVID-19 pandemic, outpatient exposure of ACEIs/ARBs were associated with the increase in poor outcomes (Jeffery et al., 2022). Our previous retrospective cohort study found that pre-hospital ACEI and ARB exposure was associated with better outcomes and acted as an independent factor for patients with acute respiratory failure (Fang and Zhang, 2022). Chronic prescription and pre-hospital β-blockers exposure can reduce ICU mortality among patients with sepsis (Macchia et al., 2012; Singer et al., 2017). Similar result has been obtained in patients with acute respiratory failure (Noveanu et al., 2010). Numerous previous studies have demonstrated the beneficial effects of CCBs in patients with sepsis (Wiewel et al., 2017) and pneumonia (Zheng et al., 2017). In a chronic obstructive pulmonary disease (COPD) cohort, the exposure of amlodipine, one kind of CCBs, was associated with the lower risk of all-causes 1-year mortality but no significant difference in the risk of severe AECOPD was found compared with Bendroflumethiazide, one kind of diuretics (Rastoder et al., 2023). Diuretics are considered the most cost-effective antihypertensive drugs (Geroy, 2012), but are not the first choice in most countries and populations (Smith et al., 2020). Administration of diuretics within 48 h of ICU admission has been shown to be related to a reduce in the incidence of positive fluid balance in septic patients with left ventricular dysfunction, but does not significantly decrease mortality (Jones et al., 2022). However, a cohort study of Chinese hypertensive patients in Hong Kong revealed that thiazide-like diuretics exhibited the greatest risk reduction for all-cause and cardiovascular mortality (Jiang et al., 2009). Oral antihypertensive drugs and good blood pressure control are known to improve overall survival in patients with hypertension (Staessen et al., 2000; Emrich et al., 2019). However, there is still a lack of sufficient evidence to explore the effect of different pre-admission antihypertensive drug regimens on the outcome of patients in critical care units (ICU).

In present study, we aimed to investigate the protective effect of pre-admission single and combined antihypertensive drug regimens on the clinical outcomes in critically ill patients using data from the Medical Information Mart for Intensive Care IV (MIMIC-IV) database (Johnson et al., 2021).

## 2 Methods

### 2.1 Data access and extraction

The present retrospective observational study utilized data from MIMIC-IV database, which is provided by Beth Israel Deaconess Medical Center (Boston, MA). MIMIC-IV, the latest version of the MIMIC database, contains 73,141 hospitalization records for 50,934 patients admitted to the ICU departments at Beth Israel Deaconess Medical Center from 2008 to 2019. The data were extracted by Yipeng Fang, who has been certified to access the research database and has completed a National Institutes of Health web-based course (certification No. 43025968). All data were extracted using the PgAdmin4 and PostgreSQL (version 9.6) software. Ethical approval for the MIMIC project was obtained through the institutional review boards of the Massachusetts Institute of Technology. Due to no additional data used in preset analysis, additional ethical approval and informed consent can be exempted. The manuscript was prepared according to the STROBE reporting checklist (Husain et al., 2015).

### 2.2 Study population

A total of 73,141 ICU admission records have been found in the MIMIC IV database. For patients with multiple admissions, only their first ICU admission records were included in the analysis. Since present study is focused on the efficacy of antihypertensive drugs, only the hypertensive population was included in the final analysis. Meanwhile, patients who met the following criteria were excluded: (1) age <18 years; (2) death within the first 24 h of their ICU admission; (3) without antihypertensive exposure before their ICU admission.

### 2.3 Baseline characteristics extraction

The baseline characteristics of patients were either obtained directly or calculated indirectly using the admission table, patients table and the ICU-detailed materialized view. Information on laboratory parameters, vital signs, disease severity scores and special interventions (including continuous renal replacement therapy (CRRT), invasive ventilation and vasoactive medications) within the first 24 h of ICU admission were obtained from the materialized views. Comorbidities were identified according to International Classification of Diseases (ICD) codes and the Charlson comorbidity index materialized view. Details of the ICD codes used to identify hypertension and comorbidities were shown in Supplementary Table S1. Both ICD-9 and ICD-10 were used in present study.

### 2.4 Exposure and outcome

The primary exposure was the consumption of oral antihypertensive medication by patients prior to their admission to the ICU departments. Intravenous anti-hypertensive drugs are indicative of emergency conditions or poor blood pressure management, which may indicate poor clinical outcomes. However, due to the unavailability of intravenous formulations for certain classes of antihypertensive drugs, all data pertaining to the intravenous administration of antihypertensive medications were excluded. Medication records for antihypertensive drugs were retrieved from the prescription system through keyword filtering. Details of the keywords for antihypertensive drugs were shown in Supplementary Table S2. In the present study, the medication records of patients prior to their ICU admission were retained as a basis for the use of antihypertensive drugs before ICU admission.

The primary outcome was 90-day mortality, and secondary outcomes included 28-day mortality, in-hospital mortality, ICU mortality, length of hospital stay (hospital-LOS) and ICU-LOS. We identified all-cause deaths within 28-day and 90-day when death occurred less than 28 and 90 days after ICU admission, according to the ICU admission time and date of death. In-hospital and ICU deaths were identified if the date of death was earlier than the hospital discharge time or the ICU out time of ICU department, respectively. Hospital-LOS (ICU-LOS) was calculated from admission to discharge time (our-time of ICU department).

### 2.5 Management of abnormal values and missing data

Abnormal values were adjusted as missing according to the two-way scatter plots. Indicators with missing percentages >5% were excluded from our final analysis. We used mean or median to replace the missing values when the percentage of missing data was <5%.

### 2.6 Statistical analysis

Normally distributed variables were expressed as mean ± standard deviation (SD), and analyzed using either Student’s t-test or Bonferroni test in ANOVA. Non-normally distributed variables were presented as median with first to third quartiles, and further compared between groups using either Mann-Whitney U or Kruskal-Wallis tests. Categorical variables were presented as numbers and proportions, and analyzed using either Chi-square or Fishers exact tests. Bonferroni test was applied to adjust the *p*-value.

Propensity score matching (PSM) and inverse probability of treatment weighting (IPTW) methods were used to balance the distribution of baseline characteristics. Generalized linear models were constructed to calculate the propensity scores (PS) with 90-day mortality as the dependent variable. The independent variables included in the these generalized linear models were demographic information (age, sex, race, weight), laboratory parameters (serum white blood cells, hemoglobin, platelets, creatinine, sodium, potassium), vital signs [temperature, heart rate, respiratory rate, mean blood pressure and percutaneous arterial oxygen saturation (SpO_2_)], comorbidities (coronary heart disease, heart failure, diabetes mellitus, chronic kidney disease, liver disease, chronic pulmonary disease, malignant cancer), disease severity score (Sequential organ failure assessment scores [SOFA] and the Simplified Acute Physiology Score II [SAPSII] scores) and special interventions (CRRT, invasive ventilation and vasoactive medications). In PSM, a 1:1 nearest neighbor no-replacement matching with a caliper width of 0.02 was used to reduce bias. In IPTW, standard weights were calculated based on PS. The probability of patients belonging to two contrasting groups is PS and 1-PS, respectively. After using the inverse of the PS for patients to calculate the weight, we obtained 1/PS and 1/(1-PS), which can be used to represent the IPTW weighs for two contrasting groups. Baseline information after matching was further compared to investigate the effect of matching. Logistic regression analysis was used to further detect the protective effect of different antihypertensive regimens. The above independent variables enrolled in the calculation of PS were applied sequentially in the logistic regression models to adjust models. It should be noted that regression estimates with IPTWs lead to an increase in the sample size and tend to reject the null hypothesis too frequently because of inflated sample sizes, which may lead to some false positive errors. The increase in sample size occurs because the IPTW assigns a weight to each patient in order to balance the baseline characteristics. Several subgroup analyses were performed to assess the robustness of our findings and to search for potential interactive factors.

All statistical analyses were performed using Stata (version 15.0) and R (version 4.1.3) software. A two-tailed test was performed, and data followed by *p* < 0.05 were considered statistically significant.

## 3 Results

### 3.1 Effect of single and combined antihypertensives exposure

**FIGURE 1:**
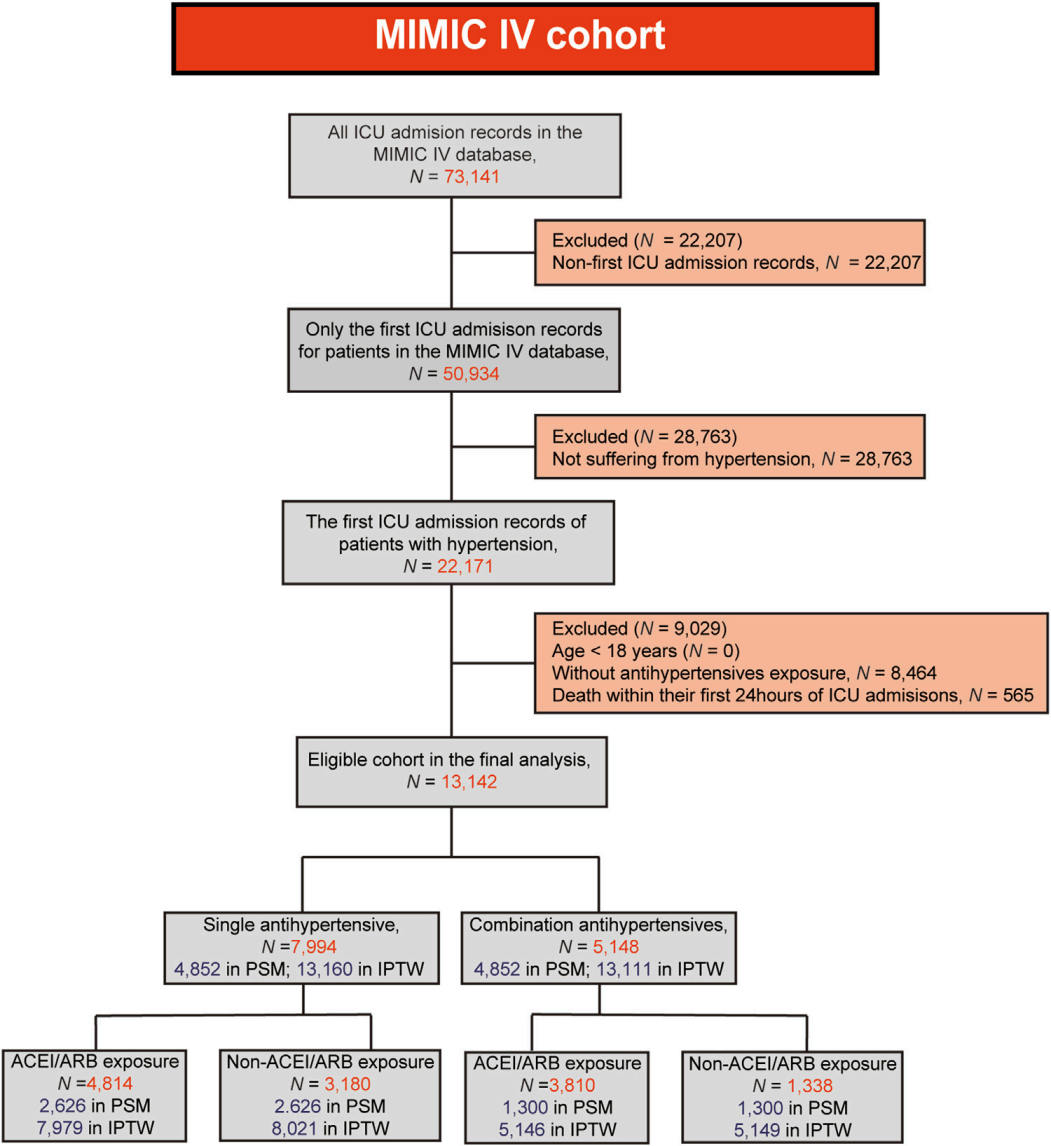
Flow chart shows patient selection and study design.

A flow chart of the present study was shown in Figure 1. A total of 13,142 patients were enrolled in the final analysis, including 7,944 with single antihypertensive drug exposure before ICU and 5,148 with combined exposure. The comparison of baseline information and clinical outcomes in the original, PSM and IPTW cohorts were shown in Table 1. In the original cohort, significant differences were present in baseline characteristics, but no significant differences in mortality indicators (all *p* > 0.05). Patients with combined therapy had a lower 90-day mortality rate than those with single antihypertensives exposure, but no statistical significance was found in original cohort (10.94% vs 11.12%, *p* = 0.742). In both the PSM and IPTW cohorts, there were no significant differences in baseline data (all *p* > 0.05). Patients with combined exposure had significantly lower 90-day and 365-day mortality compared to the single exposure groups in both the PSM and IPTW cohorts (all *p* < 0.05). The 28-day, in-hospital and ICU mortality rates were lower in patients with combined exposure, but no statistical significances were found in either PSM or IPTW cohorts (all *p* > 0.05).

**TABLE 1:** Comparison of baseline information and outcomes in patients with single and combined antihypertensive exposure.

	Original cohort			PSM cohort			IPTW cohort		
Variable	Single exposure	Combined exposures	*p*-value	Single exposure	Combined exposures	*p*-value	Single exposure	Combined exposures	*p*-value
Number	7,994	5,148		4,852	4,852		13,160	13,111	
Age (years)	68.33 ± 13.28	71.19 ± 12.52	**<0.001**	70.88 ± 12.58	70.81 ± 12.57	0.805	69.49 ± 13.11	69.55 ± 12.96	0.814
Male (%)	4,690 (58.67)	2,693 (52.31)	**<0.001**	2,582 (53.22)	2,605 (53.69)	0.640	7,391 (56.17)	7,343	0.857
Ethnicity, white (%)	5,368 (67.15)	3,437 (66.76)	0.645	3,280 (67.60)	3,254 (67.07)	0.574	8,819 (67.01)	8,804 (67.15)	0.874
Weight (kg)	83.26 ± 21.79	82.86 ± 22.38	0.313	82.67 ± 22.27	82.63 ± 22.18	0.927	83.09 ± 22.04	83.13 ± 22.19	0.919
Comorbidities									
Coronary heart disease (%)	1,936 (24.22)	1,595 (30.98)	**<0.001**	1,433 (29.53)	1,420 (29.27)	0.772	3,553 (27.00)	3,545 (27.04)	0.965
Heart failure (%)	1,215 (15.20)	1,235 (23.99)	**<0.001**	1,026 (21.15)	1,010 (20.82)	0.690	2,481 (18.85)	2,466 (18.81)	0.954
Diabetes mellitus (%)	2,531 (31.66)	1,928 (37.45)	**<0.001**	1,765 (36.38)	1,753 (36.13)	0.800	4,472 (33.98)	4,475 (34.13)	0.861
Chronic kidney disease (%)	44 (0.55)	41 (0.80)	0.086	33 (0.68)	34 (0.70)	0.902	86 (0.66)	85 (0.65)	0.936
Liver disease (%)	652 (8.16)	333 (6.47)	**<0.001**	341 (7.03)	323 (6.66)	0.469	984 (7.48)	991 (7.56)	0.877
Chronic pulmonary disease (%)	1,786 (22.34)	1,433 (27.84)	**<0.001**	1,257 (26.28)	1,270 (26.17)	0.908	3,240 (24.62)	3,231 (24.64)	0.981
Malignant cancer (%)	974 (12.18)	685 (13.31)	0.059	654 (13.48)	648 (13.36)	0.858	1,687 (12.82)	1,700 (12.97)	0.809
Vital sign									
Heart rate, bpm	86.13 ± 18.74	86.33 ± 19.22	0.550	86.27 ± 18.47	86.23 ± 19.22	0.919	86.25 ± 18.70	86.21 ± 19.27	0.920
Respiratory rate, bpm	18.26 ± 5.72	18.16 ± 5.82	0.358	18.23 ± 5.76	18.22 ± 5.86	0.899	18.24 ± 5.73	18.24 ± 5.86	0.982
Mean blood pressure, mmHg	83.90 ± 18.08	83.84 ± 17.92	**<0.001**	84.06 ± 17.94	84.18 ± 17.95	0.738	85.08 ± 18.07	85.04 ± 18.14	0.908
Temperature,°C	36.68 ± 0.72	36.64 ± 0.72	**0.002**	36.64 ± 0.73	36.64 ± 0.72	0.902	36.66 ± 0.72	36.66 ± 0.72	0.829
SpO_2_,%	98 (96,100)	99 (96,100)	**<0.001**	98 (96,100)	99 (96,100)	0.307	98 (96,100)	98 (96,100)	0.137
Disease severity score									
SOFA score	3 (2.6)	4 (2.6)	**0.001**	4 (2.6)	4 (2.6)	0.720	4 (2.6)	4 (2.6)	0.676
SAPSII score	32 (25.40)	34 (27.41)	**<0.001**	33 (27.41)	33 (27.41)	0.783	33 (26.40)	33 (26.40)	0.849
Laboratory parameters									
WBC (k/uL)	10.5 (7.8.14.3)	10.6 (7.7.14.3)	0.801	10.5 (7.7.14.1)	10.6 (7.7.14.3)	0.403	10.5 (7.8.14.2)	10.6 (7.7.14.3)	0.922
Hemoglobin (g/dL)	11.19 ± 2.23	10.57 ± 2.15	**<0.001**	10.65 ± 2.14	10.69 ± 2.11	0.310	10.94 ± 2.23	10.93 ± 2.20	0.870
Platelets (k/uL)	192 (143,250)	192 (141,251)	0.934	192 (140,251)	192 (141,251)	0.613	192 (142,251)	192 (141,249)	0.719
Creatinine (mg/dL)	0.9 (0.7.1.1)	0.9 (0.7.1.1)	**0.015**	0.9 (0.7.1.1)	0.9 (0.7.1.1)	0.166	0.9 (0.7.1.1)	0.9 (0.7.1.1)	0.274
Sodium (mmol/L)	137.68 ± 4.83	137.08 ± 4.94	**<0.001**	137.18 ± 4.93	137.19 ± 4.89	0.934	137.43 ± 4.99	137.45 ± 4.87	0.770
Potassium (mmol/L)	4.19 ± 0.69	4.20 ± 0.72	0.322	4.19 ± 0.69	4.20 ± 0.72	0.626	4.19 ± 0.70	4.19 ± 0.72	0.956
Special interventions									
Continuous renal replacement therapy (%)	54 (0.68)	27 (0.52)	0.280	28 (0.58)	27 (0.56)	0.892	81 (0.62)	83 (0.63)	0.925
Invasive ventilation (%)	2,720 (34.03)	1,820 (35.35)	0.118	1,698 (35.00)	1,691 (34.85)	0.882	4,543 (34.52)	4,507 (34.37)	0.871
Vasoactive agent (%)	1,064 (13.31)	789 (15.33)	0.001	739 (15.23)	723 (14.90)	0.650	255 (1.94)	251 (1.91)	0.916
Clinical outcomes									
365-day mortality (%)	1,419 (17.75)	943 (18.32)	0.409	991 (20.42)	871 (17.95)	**0.002**	2,486 (18.89)	2,265 (17.28)	**0.023**
90-day mortality (%)	889 (11.12)	563 (10.94)	0.742	614 (12.65)	523 (10.78)	**0.004**	1,566 (11.90)	1,356 (10.34)	**0.007**
28-day mortality (%)	564 (7.06)	364 (7.07)	0.973	383 (7.89)	338 (6.97)	0.082	992 (7.54)	890 (6.79)	0.119
Hospital mortality (%)	349 (4.37)	236 (4.58)	0.533	240 (4.95)	226 (4.66)	0.506	612 (4.65)	576 (4.39)	0.506
ICU mortality (%)	226 (2.83)	153 (2.97)	0.628	153 (3.15)	143 (2.95)	0.555	399 (3.03)	379 (2.89)	0.661
Hospital LOS (days)	6.7 (4.3.10.8)	7.3 (4.8.11.6)	**<0.001**	7.0 (4.6.10.9)	7.2 (4.8.11.2)	**0.011**	6.8 (4.4.10.9)	7.1 (4.7.11.0)	**0.004**
ICU LOS (days)	1.9 (1.1.3.5)	1.8 (1.1.3.2)	**0.003**	1.9 (1.1.3.4)	1.8 (1.1.3.2)	**<0.001**	1.9 (1.1.3.5)	1.8 (1.1.3.2)	**<0.001**

Continuous variables are displayed as mean (standard deviation) or median (first quartile–third quartile); categorical variables are displayed as count (percentage); ICU, intensive care unit; IPTW: inverse probability weighting; LOS, length of stays; PSM, propensity score matching; SAPS, simplified acute physiology score; SOFA, sequential organ failure assessment; SpO_2_, oxygen saturation; WBC, white blood cells. The expansion of the sample size occurs in the IPTW cohorts due to the IPTW method assigning each patient a certain weight to achieve balance in baseline characteristic.

Bold values indicate that there are statistically significant differences between the groups, with *p* < 0.05.

### 3.2 Therapeutic efficacy of ACEIs and ARBs in single and combined therapy cohorts

Baseline information and clinical outcomes of patients in each single antihypertensive subgroup were outlined in Table 2. In summary, we found significant differences in all baseline characteristics (all *p* < 0.05), except for chronic kidney disease (*p* = 0.431). All clinical outcomes were significantly different among all subgroups (all *p* < 0.001). In terms of 90-day mortality, patients with pre-admission ACEIs exposure (6.93%) had the lowest mortality rate, followed by those with ARBs exposure (11.43%) and β-blockers (16.18%). The similar trends were existed in all mortality indictors.

**TABLE 2:** Comparison of baseline information and outcomes in patients with different kinds of single antihypertension exposure.

Variable	ACEIs	ARBs	β-blockers	DHP-CCBs	NDHP-CCBs	Thiazide diuretics	*p*-value
Number	4,534	280	2,386	194	297	303	
Age (years)	67.59 ± 13.56	69.26 ± 12.22	69.09 ± 12.70	74.46 ± 12.21	70.37 ± 13.76	66.66 ± 13.13	**<0.001**
Male (%)	2,696 (59.46)	136 (48.57)	1,504 (63.03)	80 (41.24)	149 (50.17)	125 (41.25)	**<0.001**
Ethnicity, white (%)	2,942 (64.89)	188 (97.14)	1,705 (71.46)	142 (73.20)	202 (68.01)	189 (62.38)	**<0.001**
Weight (kg)	84.65 ± 22.38	84.96 ± 23.47	82.02 ± 20.03	75.15 ± 19.63	77.52 ± 19.75	82.71 ± 22.59	**<0.001**
Comorbidities							
Coronary heart disease (%)	885 (19.52)	51 (18.21)	910 (38.14)	28 (14.43)	36 (12.12)	26 (8.58)	**<0.001**
Heart failure (%)	745 (16.43)	46 (16.43)	338 (14.17)	33 (17.01)	34 (11.45)	19 (6.27)	**<0.001**
Diabetes mellitus (%)	1,546 (34.10)	108 (38.57)	693 (29.04)	52 (26.80)	61 (20.54)	71 (23.43)	**<0.001**
Chronic kidney disease (%)	18 (0.40)	2 (0.71)	19 (0.52)	1 (0.52)	2 (0.67)	2 (0.66)	0.431
Liver disease (%)	310 (6.84)	22 (7.86)	261 (10.95)	6 (3.09)	26 (8.75)	27 (8.91)	**<0.001**
Chronic pulmonary disease (%)	961 (21.20)	74 (26.43)	511 (21.42)	77 (39.69)	81 (27.27)	82 (27.06)	**<0.001**
Malignant cancer (%)	417 (9.20)	45 (16.07)	345 (14.46)	38 (19.59)	63 (21.21)	66 (21.78)	**<0.001**
Vital sign							
Heart rate, bpm	85.54 ± 18.61	88.26 ± 20.60	86.21 ± 18.45	89.45 ± 20.31	87.33 ± 18.74	89.02 ± 19.55	**<0.001**
Respiratory rate, bpm	18.39 ± 5.42	18.33 ± 5.57	17.77 ± 6.06	20.24 ± 6.44	18.42 ± 6.06	18.68 ± 6.26	**<0.001**
Mean blood pressure, mmHg	88.52 ± 18.50	84.25 ± 18.52	81.04 ± 16.38	84.63 ± 16.02	87.81 ± 16.90	85.62 ± 18.50	**<0.001**
Temperature,°C	36.72 ± 0.70	36.75 ± 0.75	36.58 ± 0.75	36.59 ± 0.72	36.70 ± 0.77	36.80 ± 0.67	**<0.001**
SpO_2_,%	98 (96,100)	98 (95,100)	99 (96,100)	97 (94,100)	98 (95,100)	98 (96,100)	**<0.001**
Disease severity score							
SOFA score	3 (2.5)	3 (1.6)	4 (1.6)	3 (2.5)	3 (2.6)	3 (1.5)	**<0.001**
SAPSII score	31 (24.38)	31 (25.40)	34 (28.42)	34 (27.43)	32 (26.42)	31 (24.40)	**<0.001**
Laboratory parameters							
WBC (k/uL)	10.5 (7.9.13.8)	10.5 (7.3.14.1)	11.0 (7.8.14.9)	11.1 (7.6.15.4)	10.0 (7.3.13.6)	10.5 (7.7.14.0)	**0.003**
Hemoglobin (g/dL)	11.66 ± 2.18	10.92 ± 2.08	10.47 ± 2.16	10.64 ± 2.07	10.60 ± 2.05	11.00 ± 2.17	**<0.001**
Platelets (k/uL)	199 (154,254)	205 (147,270)	169 (124,223)	207 (148,273)	202 (144,270)	217 (168,287)	**<0.001**
Creatinine (mg/dL)	0.9 (0.7.1.1)	0.9 (0.7.1.1)	0.8 (0.7.1.1)	0.8 (0.6.1.0)	0.9 (0.7.1.1)	0.9 (0.7.1.1)	**<0.001**
Sodium (mmol/L)	138.10 ± 4.81	137.64 ± 4.17	136.91 ± 4.65	137.91 ± 5.20	137.44 ± 5.47	137.49 ± 5.33	**<0.001**
Potassium (mmol/L)	4.13 ± 0.67	4.18 ± 0.72	4.33 ± 0.72	4.17 ± 0.66	4.12 ± 0.71	4.04 ± 0.67	**<0.001**
Special interventions							
Continuous renal replacement therapy (%)	20 (0.44)	3 (1.07)	26 (1.09)	2 (1.03)	2 (0.67)	1 (0.33)	**0.044**
Invasive ventilation (%)	1,459 (32.18)	77 (27.50)	963 (40.36)	62 (31.96)	87 (29.29)	72 (23.76)	**<0.001**
Vasoactive agent (%)	588 (12.31)	33 (11.79)	373 (15.63)	21 (10.82)	36 (12.12)	43 (14.19)	**0.004**
Clinical outcomes							
365-day mortality (%)	605 (13.34)	59 (21.07)	538 (22.55)	59 (30.41)	79 (26.60)	79 (26.07)	**<0.001**
90-day mortality (%)	314 (6.93)	32 (11.43)	386 (16.18)	45 (23.20)	51 (17.17)	61 (20.13)	**<0.001**
28-day mortality (%)	174 (3.84)	17 (6.07)	265 (11.11)	33 (17.01)	31 (10.44)	44 (14.52)	**<0.001**
Hospital mortality (%)	94 (2.07)	10 (3.57)	176 (7.38)	23 (11.86)	21 (7.07)	25 (8.25)	**<0.001**
ICU mortality (%)	45 (0.99)	9 (3.21)	119 (4.99)	14 (7.22)	16 (5.39)	23 (7.59)	**<0.001**
Hospital LOS (days)	6.2 (3.9.10.6)	6.5 (4.0.10.0)	7.6 (5.1.11.1)	8.0 (4.7.12.1)	7.0 (4.0.11.8)	6.0 (3.4.11.1)	**<0.001**
ICU LOS (days)	2.0 (1.2.3.9)	1.9 (1.1.3.1)	1.8 (1.1.3.0)	1.9 (1.1.3.4)	1.8 (1.0.3.7)	1.7 (1.0.3.0)	**<0.001**

Continuous variables are displayed as mean (standard deviation) or median (first quartile–third quartile); categorical variables are displayed as count (percentage); DHP-CCBs, dihydropyridine calcium channel blockers; ICU, intensive care unit; IPTW, inverse probability weighting; LOS, length of stays; NDHP-CCBs, non-dihydropyridine calcium channel blockers; PSM, propensity score matching; SAPS, simplified acute physiology score; SOFA, sequential organ failure assessment; SpO_2_, oxygen saturation; WBC, white blood cells.

Bold values indicate that there are statistically significant differences between the groups, with p < 0.05.

As ACEIs and ARBs have similar pharmacological and superior protective effects, they were combined into one group in the further study. We divided patients with only one type of antihypertensive exposure into two groups, based on whether they used ACEIs/ARBs: ACEIs/ARBs (+) and ACEIs/ARBs (−). Significant differences were found in terms of baseline information between the ACEIs/ARBs (+) and the ACEIs/ARBs (−) subgroups (all *p* < 0.05), except for sex (*p* = 0.722, shown in Table 3). Patients with pre-admission ACEIs/ARBs exposure had significantly decreased mortality rates compared with patients in the ACEIs/ARBs (−) subgroup (all *p* < 0.001). The PSM and IPTW methods significantly reduced the difference of baseline characteristics between the ACEIs/ARBs (+) and the ACEIs/ARBs (−) subgroups. In the PSM cohort, there were no significant differences in the baseline characteristics except for hemoglobin (*p* = 0.037) and serum creatinine (*p* = 0.002). In the IPTW cohort, 8,021 observations with pre-admission ACEIs/ARBs exposure and 7,979 without ACEIs/ARBs exposure were enrolled after weighting, and significant difference was found only for serum creatinine (*p* = 0.001) in all baseline characteristics. In both the PSM and IPTW cohorts, patients with pre-admission ACEIs/ARBs exposure had significantly lower mortality rates for all morality indicators (all *p* < 0.001).

**TABLE 3:** Comparison of baseline information and outcomes with and without ACEI/ARB exposure in the single exposure cohort.

	Original cohort			PSM cohort			IPTW cohort		
Variable	ACEI/ARB (−)	ACEI/ARB (+)	*p*-value	ACEI/ARB (−)	ACEI/ARB (+)	*p*-value	ACEI/ARB (−)	ACEI/ARB (+)	*p*-value
Number	3,180	4,814		2,626	2,626		7,979	8,021	
Age (years)	69.30 ± 12.90	67.69 ± 13.49	**<0.001**	68.99 ± 13.21	69.46 ± 13.09	0.199	68.53 ± 13.40	68.45 ± 13.20	0.811
Male (%)	1,858 (58.43)	2,832 (58.83)	0.722	1,498 (57.04)	1,487 (56.63)	0.759	4,555 (57.09)	4,655 (58.03)	0.449
Ethnicity, white (%)	2,238 (70.38)	3,130 (65.02)	**<0.001**	1,822 (69.38)	1,845 (70.26)	0.489	5,382 (67.45)	5,395 (67.26)	0.872
Weight (kg)	81.24 ± 20.33	84.67 ± 22.45	**<0.001**	81.96 ± 20.72	81.38 ± 20.66	0.316	83.22 ± 21.81	82.12 ± 21.86	0.862
Comorbidities									
Coronary heart disease (%)	1,000 (31.45)	936 (19.44)	**<0.001**	689 (26.24)	733 (27.91)	0.172	1,916 (24.01)	1,935 (24.12)	0.920
Heart failure (%)	424 (13.33)	791 (16.43)	**<0.001**	372 (14.17)	389 (14.81)	0.505	1,203 (15.09)	1,220 (15.21)	0.893
Diabetes mellitus (%)	877 (27.58)	1,654 (34.36)	**<0.001**	774 (29.47)	759 (28.90)	0.649	2,524 (31.63)	2,518 (31.39)	0.834
Chronic kidney disease (%)	24 (0.75)	20 (0.42)	**0.045**	16 (0.61)	15 (0.57)	0.857	43 (0.51)	45 (0.56)	0.920
Liver disease (%)	320 (10.06)	332 (6.90)	**<0.001**	232 (8.83)	216 (8.23)	0.429	604 (7.57)	631 (7.86)	0.642
Chronic pulmonary disease (%)	751 (23.62)	1,035 (21.50)	**0.026**	623 (23.72)	638 (24.30)	0.628	1,841 (23.08)	1,831 (22.83)	0.814
Malignant cancer (%)	512 (16.10)	462 (9.60)	**<0.001**	360 (13.71)	369 (14.05)	0.719	976 (12.24)	999 (12.46)	0.782
Vital sign									
Heart rate, bpm	86.78 ± 18.72	85.70 ± 18.74	**0.011**	86.15 ± 18.64	86.28 ± 18.51	0.809	85.89 ± 18.50	86.10 ± 18.66	0.643
Respiratory rate, bpm	18.07 ± 6.13	18.39 ± 5.43	**0.015**	18.19 ± 6.10	18.11 ± 5.50	0.620	18.27 ± 6.09	18.24 ± 5.51	0.847
Mean blood pressure, mmHg	82.33 ± 16.77	88.27 ± 18.52	**<0.001**	84.01 ± 17.05	83.62 ± 17.21	0.408	85.70 ± 17.94	85.80 ± 18.25	0.832
Temperature,°C	36.61 ± 0.74	36.72 ± 0.70	**<0.001**	36.66 ± 0.71	36.64 ± 0.74	0.370	36.67 ± 0.71	36.68 ± 0.73	0.914
SpO_2_,%	99 (96,100)	98 (96,100)	**<0.001**	99 (96,100)	98 (96,100)	0.355	98 (96,100)	98 (96,100)	0.322
Disease severity score									
SOFA score	4 (2.6)	3 (2.5)	**<0.001**	4 (2.6)	4 (2.6)	0.621	3 (2.6)	3 (2.6)	0.537
SAPSII score	34 (27.42)	31 (24.38)	**<0.001**	33 (26.41)	33 (27.41)	0.172	32 (25.40)	32 (25.40)	0.777
Laboratory parameters									
WBC (k/uL)	10.7 (7.7.14.8)	10.5 (7.9.13.9)	**0.046**	10.6 (7.6.14.4)	10.5 (7.9.14.5)	0.421	10.5 (7.7.14.3)	10.5 (7.8.14.1)	0.781
Hemoglobin (g/dL)	10.54 ± 2.15	11.62 ± 2.18	**<0.001**	10.87 ± 2.10	10.75 ± 2.06	**0.037**	11.16 ± 2.22	11.16 ± 2.29	0.897
Platelets (k/uL)	178 (129,238)	199 (153,255)	**<0.001**	185 (134,246)	190 (141,244)	0.317	192 (145,249)	193 (145,249)	0.391
Creatinine (mg/dL)	0.8 (0.7.1.1)	0.9 (0.7.1.1)	**<0.001**	0.9 (0.7.1.1)	0.9 (0.7.1.1)	**0.002**	0.9 (0.7.1.1)	0.9 (0.7.1.1)	**0.001**
Sodium (mmol/L)	137.08 ± 4.84	138.07 ± 4.78	**<0.001**	137.49 ± 4.81	137.39 ± 5.00	0.451	137.73 ± 4.85	137.70 ± 5.04	0.784
Potassium (mmol/L)	4.27 ± 0.72	4.14 ± 0.67	**<0.001**	4.22 ± 0.69	4.22 ± 0.71	0.958	4.18 ± 0.68	4.19 ± 0.70	0.874
Special interventions									
Continuous renal replacement therapy (%)	31 (0.97)	23 (0.48)	**0.008**	20 (0.76)	19 (0.72)	0.872	55 (0.68)	57 (0.71)	0.891
Invasive ventilation (%)	1,184 (37.23)	1,536 (31.91)	**<0.001**	922 (35.11)	920 (35.03)	0.954	2,681 (33.60)	2,739 (34.15)	0.638
Vasoactive agent (%)	473 (14.87)	591 (12.28)	**0.001**	371 (14.13)	371 (14.13)	1.000	164 (2.06)	154 (1.92)	0.699
Clinical outcomes									
365-day mortality (%)	755 (23.74)	644 (13.79)	**<0.001**	601 (22.89)	420 (15.99)	**<0.001**	1,711 (21.44)	1,180 (14.71)	**<0.001**
90-day mortality (%)	543 (17.08)	346 (7.19)	**<0.001**	422 (16.07)	221 (8.42)	**<0.001**	1,204 (15.09)	615 (7.67)	**<0.001**
28-day mortality (%)	373 (11.73)	191 (3.97)	**<0.001**	283 (10.78)	115 (4.38)	**<0.001**	810 (10.15)	329 (4.11)	**<0.001**
Hospital mortality (%)	245 (7.70)	104 (2.16)	**<0.001**	185 (7.04)	58 (2.21)	**<0.001**	536 (6.71)	184 (2.29)	**<0.001**
ICU mortality (%)	172 (5.41)	54 (1.12)	**<0.001**	136 (5.18)	31 (1.18)	**<0.001**	378 (4.74)	94 (1.18)	**<0.001**
Hospital LOS (days)	7.3 (4.9.11.2)	6.2 (3.9.10.6)	**<0.001**	7.1 (4.8.11.0)	6.5 (4.2.10.8)	**<0.001**	6.9 (4.5.10.8)	6.4 (4.1.10.7)	**0.004**
ICU LOS (days)	1.8 (1.1.3.1)	2.0 (1.2.3.9)	**<0.001**	1.8 (1.1.3.1)	2.0 (1.1.3.8)	**<0.001**	1.8 (1.1.3.0)	2.0 (1.2.3.9)	**<0.001**

Continuous variables are displayed as mean (standard deviation) or median (first quartile–third quartile); categorical variables are displayed as count (percentage); ICU, intensive care unit; IPTW: inverse probability weighting; LOS, length of stays; PSM, propensity score matching; SAPS, simplified acute physiology score; SOFA, sequential organ failure assessment; SpO_2_, oxygen saturation; WBC, white blood cells. The expansion of the sample size occurs in the IPTW cohorts due to the IPTW method assigning each patient a certain weight to achieve balance in baseline characteristic.

Bold values indicate that there are statistically significant differences between the groups, with p < 0.05.

The protective effect of ACEIs and ARBs was further detected in the combined therapy cohort. We found 3,810 in the combining-ACEIs/ARBs (+) and 1,338 the combining-ACEIs/ARBs (−) subgroups, respectively. In the original cohort, patients in the combining-ACEIs/ARBs (+) subgroup had significantly lower mortality rates than patients in the combining-ACEIs/ARBs (−) subgroup (all *p* < 0.001, Table 4). In the PSM and IPTW matched cohort, combining-ACEIs/ARBs (+) therapy was also associated with a significant reduction in all mortality indicators (all *p* ≤ 0.001, shown in Table 4).

**TABLE 4:** Comparison of baseline information and outcomes with and without ACEI/ARB exposure in the combined therapy cohort.

	Original cohort			PSM cohort			IPTW cohort		
Variable	Combining-ACEI/ARB (−)	Combining-ACEI/ARB (+)	*p*-value	Combining-ACEI/ARB (−)	Combining-ACEI/ARB (+)	*p*-value	Combining-ACEI/ARB (−)	Combining-ACEI/ARB (+)	*p*-value
Number	1,338	3,810		1,300	1,300		5,149	5,146	
Age (years)	71.92 ± 12.83	70.93 ± 12.40	**0.012**	71.91 ± 12.86	72.16 ± 12.57	0.609	71.07 ± 12.85	71.15 ± 14.44	0.847
Male (%)	649 (48.51)	2,044 (53.65)	0.001	634 (48.77)	633 (48.69)	0.969	2,726 (52.95)	2,696 (52.39)	0.740
Ethnicity, white (%)	870 (65.02)	2,567 (67.38)	0.116	848 (65.23)	837 (64.38)	0.651	3,455 (67.10)	3,437 ± 66.79	0.844
Weight (kg)	80.05 ± 21.14	83.92 ± 22.58	**<0.001**	80.37 ± 21.18	79.03 ± 20.24	0.100	82.99 ± 22.17	82.95 ± 22.28	0.966
Comorbidities									
Coronary heart disease (%)	347 (25.93)	1,248 (32.76)	**<0.001**	342 (26.31)	302 (23.23)	0.069	1,576 (30.61)	1,593 (30.95)	0.833
Heart failure (%)	241 (18.01)	994 (26.09)	**<0.001**	239 (18.38)	211 (16.23)	0.147	1,233 (23.95)	1,236 (24.02)	0.967
Diabetes mellitus (%)	375 (28.03)	1,553 (40.76)	**<0.001**	374 (28.77)	293 (22.54)	**<0.001**	1937 (37.62)	1930 (37.50)	0.941
Chronic kidney disease (%)	13 (0.97)	28 (0.7)	0.402	12 (0.92)	13 (1.00)	0.841	51 (0.99)	44 (0.85)	0.669
Liver disease	104 (7.77)	229 (6.01)	**0.024**	96 (7.38)	106 (8.15)	0.464	328 (6.38)	333 (6.48)	0.899
Chronic pulmonary disease	363 (27.13)	1,070 (28.08)	0.503	348 (26.77)	322 (24.77)	0.244	1,424 (27.66)	1,432 (27.82)	0.918
Malignant cancer	261 (19.51)	424 (11.13)	**<0.001**	230 (17.69)	261 (20.08)	0.120	668 (12.97)	678 (13.17)	0.840
Vital sign									
Heart rate, bpm	88.65 ± 20.64	85.52 ± 18.63	**<0.001**	88.25 ± 20.37	88.98 ± 20.51	0.365	86.14 ± 19.22	86.30 ± 19.11	0.795
Respiratory rate, bpl	18.68 ± 6.19	17.98 ± 5.67	**<0.001**	18.55 ± 6.15	18.65 ± 5.90	0.694	18.04 ± 5.98	18.15 ± 5.76	0.595
Mean blood pressure, mmHg	83.26 ± 17.34	84.05 ± 18.12	0.169	83.33 ± 17.24	82.74 ± 18.17	0.395	83.43 ± 11.09	83.79 ± 18.18	0.528
Temperature,°C	36.62 ± 0.72	36.64 ± 0.73	0.330	36.62 ± 0.71	36.62 ± 0.79	0.823	36.64 ± 0.69	36.64 ± 0.74	0.933
SpO_2_,%	98 (95,100)	99 (96,100)	**<0.001**	98 (95,100)	98 (95,100)	0.189	98 (96,100)	98 (96,100)	0.454
Disease severity score									
SOFA score	4 (2.6)	4 (2.6)	**<0.001**	4 (2.6)	4 (2.6)	0.377	4 (2.6)	4 (2.6)	0.670
SAPSII score	35 (28.42)	33 (26.41)	**<0.001**	35 (28.42)	35 (29.43)	0.105	34 (27.41)	34 (27.41)	0.804
Laboratory parameters									
WBC (k/uL)	10.8 (7.5.14.8)	10.5 (7.7.14.1)	0.189	10.8 (7.5.14.7)	10.6 (7.9.14.5)	0.963	10.7 (7.5.14.7)	10.6 (7.7.14.3)	0.638
Hemoglobin (g/dL)	10.43 ± 2.09	10.62 ± 2.16	**0.007**	10.47 ± 2.09	10.42 ± 2.13	0.567	10.54 ± 2.10	10.57 ± 2.16	0.636
Platelets (k/uL)	193 (140,260)	192 (141,248)	0.271	192 (140,259)	192 (135,257)	0.301	192 (141,256)	192 (140,250)	0.469
Creatinine (mg/dL)	0.9 (0.7.1.2)	0.9 (0.7.1.1)	0.273	0.9 (0.7.1.1)	0.9 (0.7.1.1)	0.897	0.9 (0.7.1.1)	0.9 (0.7.1.1)	0.647
Sodium (mmol/L)	137.28 ± 5.11	137.01 ± 4.88	0.093	137.28 ± 5.06	137.28 ± 5.03	0.991	137.10 ± 4.91	137.08 ± 4.90	0.927
Potassium (mmol/L)	4.16 ± 0.76	4.21 ± 0.70	**0.028**	4.16 ± 0.76	4.13 ± 0.67	0.302	4.20 ± 0.77	4.20 ± 0.70	0.968
Special interventions									
Continuous renal replacement therapy, %	12 (0.90)	15 (0.39)	**0.028**	9 (0.69)	11 (0.85)	0.653	33 (0.4)	28.30 (0.55)	0.704
Invasive ventilation, %	470 (35.13)	1,350 (35.43)	0.840	453 (34.85)	449 (34.54)	0.869	1837 (35.68)	1821 (35.37)	0.849
Vasoactive agent, %	208 (15.55)	581 (15.25)	0.796	201 (15.46)	202 (15.54)	0.957	104 (2.03)	104 (2.03)	0.974
Clinical outcomes									
365-day mortality	383 (28.62)	560 (14.70)	**<0.001**	358 (27.54)	249 (19.15)	**<0.001**	1,257 (24.42)	821 (15.96)	**<0.001**
90-day mortality	253 (18.91)	310 (8.14)	**<0.001**	234 (18.00)	151 (11.62)	**<0.001**	775 (15.05)	459 (8.92)	**<0.001**
28-day mortality	182 (13.60)	182 (4.78)	**<0.001**	167 (12.85)	94 (7.23)	**<0.001**	539 (10.46)	275 (5.33)	**<0.001**
Hospital mortality	130 (9.72)	106 (2.78)	**<0.001**	116 (8.92)	58 (4.46)	**<0.001**	384 (7.45)	162 (3.15)	**<0.001**
ICU mortality	100 (7.47)	53 (1.39)	**<0.001**	87 (6.69)	28 (2.15)	**<0.001**	303 (5.89)	85 (1.65)	**<0.001**
Hospital LOS	7.7 (4.8.12.0)	7.2 (4.8.11.2)	0.090	7.7 (4.8.11.9)	7.7 (5.0.12.2)	0.263	7.4 (4.7.11.7)	7.3 (4.9.11.4)	0.896
ICU LOS	1.9 (1.1.3.2)	1.8 (1.1.3.2)	0.585	1.9 (1.1.3.2)	1.9 (1.1.3.4)	0.345	1.8 (1.1.3.1)	1.9 (1.1.3.2)	0.199

Continuous variables are displayed as mean (standard deviation) or median (first quartile–third quartile); categorical variables are displayed as count (percentage); ICU, intensive care unit; IPTW: inverse probability weighting; LOS, length of stays; PSM, propensity score matching; SAPS, simplified acute physiology score; SOFA, sequential organ failure assessment; SpO_2_, oxygen saturation; WBC, white blood cells. The expansion of the sample size occurs in the IPTW cohorts due to the IPTW method assigning each patient a certain weight to achieve balance in baseline characteristic.

Bold values indicate that there are statistically significant differences between the groups, with *p* < 0.05.

### 3.3 Logistic regression model and subgroup analysis

Table 5 showed that in unadjusted Model 1 of the original cohort, no significant protective value of combined therapy was detected (OR 0.98, 95% CI 0.80–1.10, *p* = 0.742). But combined antihypertensive therapy previous ICU admission was considered as an independent protective factor for 90-day mortality in the PSM, IPWT cohorts and all adjusted models, with a 12%–20% reduction in the risk of death (OR 0.80–0.88, all *p* < 0.05).

**TABLE 5:** Logistic regression model of the protective effect of exposure to different antihypertensive regimens on the risk of 90-day mortality.

	Unadjusted model		Adjusted model 1		Adjusted model 2		Adjusted model 3	
90-day mortality	OR (95% CI)	*p*-value	OR (95% CI)	*p*-value	OR (95% CI)	*p*-value	OR (95% CI)	*p*-value
Pre-ICU combined antihypertensive exposure								
Original cohort	0.98 (0.88.1.10)	0.742	0.88 (0.79.0.99)	**0.036**	0.80 (0.71.0.91)	**<0.001**	0.80 (0.70.0.91)	**0.001**
PSM cohort	0.86 (0.76.0.97)	**0.015**	0.86 (0.76.0.98)	**0.021**	0.83 (0.72.0.95)	**0.007**	0.81 (0.70.0.93)	**0.003**
IPTW cohort	0.85 (0.80.0.92)	**<0.001**	0.85 (0.78.0.92)	**<0.001**	0.82 (0.75.0.89)	**<0.001**	0.82 (0.73.0.87)	**<0.001**
ACEI/ARB exposure in single antihypertensive cohort								
Original cohort	0.38 (0.33.0.43)	**<0.001**	0.39 (0.34.0.45)	**<0.001**	0.44 (0.37.0.51)	**<0.001**	0.45 (0.38.0.53)	**<0.001**
PSM cohort	0.48 (0.40.0.57)	**<0.001**	0.46 (0.38.0.55)	**<0.001**	0.45 (0.37.0.54)	**<0.001**	0.43 (0.36.0.52)	**<0.001**
IPTW cohort	0.47 (0.42.0.52)	**<0.001**	0.46 (0.41.0.51)	**<0.001**	0.45 (0.40.0.50)	**<0.001**	0.43 (0.38.0.48)	**<0.001**
ACEI/ARB exposure in combined antihypertensive cohort								
Original cohort	0.38 (0.32.0.45)	**<0.001**	0.40 (0.33.0.48)	**<0.001**	0.49 (0.40.0.60)	**<0.001**	0.51 (0.41.0.62)	**<0.001**
PSM cohort	0.60 (0.48.0.75)	**<0.001**	0.57 (0.46.0.72)	**<0.001**	0.51 (0.40.0.65)	**<0.001**	0.50 (0.38.0.64)	**<0.001**
IPTW cohort	0.55 (0.49.0.63)	**<0.001**	0.54 (0.48.0.62)	**<0.001**	0.52 (0.46.0.59)	**<0.001**	0.50 (0.43.0.57)	**<0.001**

Model 1 = adjusting sex, age, ethnicity and weight.

Model 2 = Model 1 + adjusting coronary heart disease, heart failure, diabetes mellitus, chronic kidney disease, liver disease, chronic pulmonary disease, malignant cancer, temperature, heart rate, respiratory rate, mean blood pressure, SpO_2,_ white blood cells, platelets, hemoglobin, sodium, potassium, creatinine.

Model 3 = Model 2 + adjusting continuous renal replacement therapy, invasive ventilation, vasoactive agent, sofa score and SAPSII score.

CI, confidence interval; IPTW, inverse probability weighting; PSM, propensity score matching; OR, odds ratio; SAPS, simplified acute physiology score; SOFA, sequential organ failure assessment.

Bold values indicate that there are statistically significant differences between the groups, with *p* < 0.05.

Pre-admission ACEIs/ARBs exposure was found to be as an independent protective factor for 90-day mortality in both single and combined therapy cohorts (all OR <1, *p* < 0.001, shown in Table 5). In the single therapy cohort, pre-admission ACEIs/ARBs exposure reduced the risk of 90-day death by 52%–62% (OR 0.38–0.48, all *p* < 0.001). In the combined therapy cohort, the use of ACEIs/ARBs before ICU was related to a decrease in the risk of 90-day death by 40%–62% in different models (OR 0.38–0.60, all *p* < 0.001).

**FIGURE 2:**
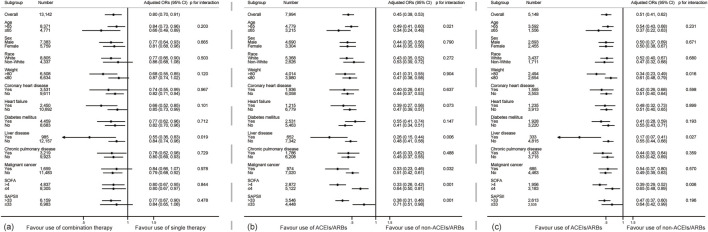
Subgroup analyses show the relationship between different drug regimens and 90-day mortality in critically ill patients. **(A)** shows the subgroup analysis about the comparing combined and single therapy **(B,C)** shows the subgroup analysis of the therapeutic effect of ACEIs/ARBs in the single and combined therapy cohorts. Adjusted ORs were adjusted by Model 3 mentioned in Table 5.

Subgroup analysis (shown in Figure 2) revealed that pre-admission combined antihypertensive therapy reduced the risk of 90-day mortality (OR < 1), but no significant difference was found in the subgroups of white race, non-white race, body weight ≤80 kg, with malignant cancer and SAPSII score ≤33 (OR < 1, *p* > 0.05) in the original cohort. There was a significant liver disease × exposure interaction (*p* for interaction = 0.019). Combined therapy reduced the risk of death more significantly in patients with liver disease (OR 0.55, 95% CI 0.36–0.83, *p* < 0.001). The results of all subgroups showed that pre-admission ACEIs/ARBs exposure was associated with the decreased risk of 90-day mortality in the single and combined therapy cohorts (all OR < 1). Although some significant interactions were observed (*p* for interaction <0.05), all adjusted ORs in those subgroups were still smaller than 1, consisting with the overall cohort. The results of subgroup analysis in the PSM and IPTW cohorts were similar to that in the original cohort (shown in Supplementary Figure S1).

## 4 Discussion

To date, only a handful of studies have elucidated the relationship between different pre-admission antihypertensive drugs and the prognosis of patients admitted to the ICU. In our present study, we retrospectively analyzed data from the MIMIC-IV database to shed light on this issue. In particular, patients on combined antihypertensive therapy had a lower 90-day mortality rate. We also found that patients with exposure to either ACEIs or ARBs had lower mortality rates than those without exposure. The protective effect of ACEIs/ARBs was present in both the single and combined exposure cohorts. Our findings were even more robust in the PSM and IPTW matched cohorts. Taken together, these results suggest that antihypertensive therapy with either ACEIs or ARBs and combined antihypertensive therapy before ICU admission are associated with decreased mortality in critically ill patients, as they are expected to confer a lower risk of mortality and are considered a protective factor.

Combined therapy is emphasized by guidelines, which not only improve the efficacy of therapy but also reduce side effects associated with treatment (Chrysant et al., 2008). Generally, combined antihypertensive therapy has been shown to effectively control blood pressure. Every 2 mmHg reduction of systolic blood pressure has been shown to markedly reduce mortality rates in patients with ischemic heart disease and stroke by 7% and 10%, respectively (Lewington et al., 2002). However, achieving the desired blood-control target via monotherapy is challenging, with previous studies showing that about 75% of hypertensive patients require at least two types of antihypertensive drugs for blood pressure control (Gradman et al., 2010). In addition, a combined of antihypertensive drugs is mainly used to suppress the side effects of single drugs (Cowart and Taylor, 2012). Furthermore, the collective pharmacological effects of ACEIs/ARBs, β-blockers, and diuretics not only decrease blood pressure but also provide additional benefits. These medications exhibit distinct mechanisms, such as the regulation of RAAS by ACEIs/ARBs, the attenuation of oxygen consumption by β-blockers, and the mitigation of volume overload by diuretics. The majority of previous studies have investigated the association of antihypertensive medications with cardio-cerebrovascular diseases, but few studies have explored the effect of antihypertensive medications in patients with critically ill disease. In this study, we found that pre-admission combined antihypertensive therapy was associated with decreased mortality rates in critically ill patients compared with those in the single antihypertensive therapy subgroup.

Another aim of this study was to determine the protective effect of different kinds of antihypertensive drugs. In particular, we found that patients who were treated with ACEIs/ARBs prior to ICU admission had significantly better outcomes. Our findings were partly consistent with the results of some previous clinical studies, although there are some differences, such as the target populations and the timing of administration. A meta-analysis including 96,159 septic patients reported that premorbid ACEIs/ARBs were significantly associated with a lower mortality but higher risk of AKI development (Hasegawa et al., 2022). A study by Zhu X et al. reported that ACEIs/ARBs exposure significantly reduced the mortality in critically ill patients but increased the higher risk of acute kidney disease compared with those without ACEIs/ARBs exposure (Zhu et al., 2022). ACEIs treatment is also linked to the lower mortality rate in patients with multiple organ dysfunction syndromes (Schmidt et al., 2010). ACEIs/ARBs have been found to exert beneficial effects in acute myocardial infarction (Zhao et al., 2022), heart failure (Cohn et al., 2001), kidney disease (Zhang et al., 2020), influenza and pneumonia cohorts (Christiansen et al., 2020).

The protective effects of ACEIs and ARBs are mainly related to the activation of the RAAS system. Firstly, these classical medications work by reducing the production of AngII and blocking the hormone receptors AT1R, thereby helping to maintain water-electrolyte balance and normal blood pressure levels, which are crucial for human health. Secondly, ACEIs/ARBs exposure reduces levels of inflammatory cytokines and preserves the barrier function (Schuett et al., 2009; Salgado et al., 2010). Since this inflammatory cascade, which could lead to disease worsening and multiple organ dysfunction in a short time, is common in the development of critical illnesses, early prophylactic and therapeutic intervention in patients with critically ill disease may be more effective. It means that ACEIs and ARBs therapy before the onset of critically diseases and in the very-early stage may be a more effective regimen in the term of anti-inflammation. Thirdly, the protective effect of ACEIs/ARBs on cardio-cerebrovascular system is another important factor. Inhibition of RAAS could improve the outcome and reduce the risk of long-term major adverse cardiac events (Ou et al., 2021; Chen et al., 2023). Fourthly, long-term exposure of ACEIs/ARBs effectively improves the expression level of ACE2 which exerts vascular protective actions (Ferrario et al., 2005; Kriszta et al., 2021). Fifthly, the rebound hyperactivity of the RAAS axis has been mentioned as a potential mechanism recently (Hasegawa et al., 2022), which is related to the upregulation of ACE and AngII receptors caused by long-term ACEIs/ARBs exposure. Through enhancing the negative feedback loop from AngII, the rebound hyperactivity may inhibit the level of renin, which has been reported as a risk factor in critically ill patients (Flannery et al., 2021). Further work is needed to explore these potential mechanisms.

Although our findings emphasized the advantage of combined therapy and ACEIs/ARBs exposure, it does not mean that combined therapy and the use of ACEIs/ARBs are necessary and inevitably beneficial. All medications should be used according to the indications and contraindications. Our present findings may encourage prescribers to consider the added benefit of combined therapy and ACEIs/ARBs. The benefit effect of long-term ACEIs/ARBs exposure is yet another important issue that needs to be considered by prescribers. What’s more, the combined antihypertensive therapy and ACEIs/ARBs exposure prior to ICU admission could be used as a protective factor and contribute to the prognosis prediction in critical ill patients.

The side effects of excessive blood control can be severe and should not be ignored, especially in elderly and frail patients. According to the PARTAGE study, combined antihypertensive therapy may increase the risk of mortality in patients with systolic blood pressure below than 130 mmHg (Benetos et al., 2015). Considering the complicated and fragile state of critically ill patients, combined antihypertensive therapy may be more effective than single antihypertensive therapy in critically ill patients, but adverse events, such as hypotension induced by excessive blood control, need attention.

This study also has some limitations, and the findings should be interpreted with caution. Firstly, the impact of single *versus* combined antihypertensive drugs on the 90-day mortality appears uncertain, as we only obtain positive results in the matched cohorts but not in original cohort. While we can explain these results through imbalances at baseline, this still diminishes our confidence in the accuracy of the conclusions. Secondly, as a retrospective study, although PSM and IPTW methods were used, the bias caused by potential confounding factors cannot be completely eliminated. Thirdly, the causal relationship between different pre-admission antihypertensive regimens and prognosis cannot be confirmed. Fourthly, our findings cannot accurately reflect the long-term prognostic impact on patients. Doherty et al. reported that patients with ICU admission have a higher mortality rate in the next 10 years after ICU discharge compared with those without ICU admission (Doherty et al., 2022); therefore, long-term follow-up studies should be performed to determine the long-term effect on critically ill patients. Fifthly, as it was not possible to obtain information on duration of medication, frequency of antihypertensive medication, whether medication was taken regularly, and blood pressure control goal, the effect of these factors on the results was not considered in this study. This might have biased the results. Sixthly, since patients receiving intravenous formulations might have more severe disease and a poorer prognosis, only oral antihypertensive records have been considered as the primary exposure would cause bias. Finally, because we used data from a single center, the generalizability of our findings remains to be investigated.

## 5 Conclusion

In this observational study of critically ill patients, the risk of death differed according to exposure to single and combined antihypertensive drugs before admission to ICU department. Compared with exposure to single antihypertensive drugs, combined therapy was associated with lower mortality in critically ill patients. Meanwhile, both ACEIs and ARBs were associated with a lower risk of death in both the single and combined antihypertensive cohorts. More high-quality clinical researches are needed to confirm our findings.

## Data Availability

Publicly available datasets were analyzed in this study. This data can be found here: The publicly available MIMIC IV (https://physionet.org/content/mimiciv/2.2/). All details of our raw data and codes we used can be obtained from the corresponding author on reasonable request.
