# Supplementary material for: Effect of different single and combined antihypertensive drug regimens on the mortality of critical care patients
Source: Front Pharmacol. 2024 Aug 28;15:1385397. doi: 10.3389/fphar.2024.1385397 (PMC11391424; doi:10.3389/fphar.2024.1385397)
Supplement: Supplementary file 2 [file DataSheet1.docx]

Supplemental material

**Effect of different single and combined** **antihypertensive drug regimens on the mortality of critical care patients**

Yipeng Fang ^1,2,3^, Xianxi Huang ^4^, Junyu Shi ^3^, Yuan Zhang ^3^, Yinlong Deng ^3^, Chunhong Ren ^5^, *Xin Zhang ^1,2,3^

1. Laboratory of Molecular Cardiology, The First Affiliated Hospital of Shantou University Medical College, Shantou, Guangdong province, People's Republic of China

2. Laboratory of Medical Molecular Imaging, The First Affiliated Hospital of Shantou University Medical College, Shantou, Guangdong province, People's Republic of China

3. Shantou University Medical College, Shantou, Guangdong province, People's Republic of China

4. Department of Cardiology, The First Affiliated Hospital of Shantou University Medical College, Shantou, Guangdong province, People's Republic of China

5. International Medical Service Center, The First Affiliated hospital of Shantou University Medical College, Shantou, Guangdong province, People's Republic of China

Corresponding author: Xin Zhang, MD, PhD, Laboratory of Molecular Cardiology, First Affiliated Hospital of Shantou University Medical College 57^th^ Changping Road, Shantou, Guangdong province, China, 515041

Email: walterzhangx@139.com

**List of Supplemental Material**

**Table S1** The ICD cords and standards used to screen for hypertension and comorbidities

**Table S2** The list of oral antihypertensive drugs included in present study

**Figure S1** The result of subgroup analyses in PSM and IPTW cohorts. (a,b) show the results of subgroup analysis in the comparing combined and single therapy. (c,d) showed the results of subgroup analyzes regarding the protective effect of ACEIs/ARBs exposure in the single antihypertensive drug exposure cohort, and (e,f) shown that the protective effect of ACEIs/ARBs in the combined therapy cohort. (a,c,e) represent results from the PSM cohorts, while (b,d,f) represent results from the IPTW cohorts**Table S1** The ICD cords and standards used to screen for hypertension and comorbidities

| Comorbidities | ICD codes |
| --- | --- |
| Hypertension (HT) | 4010, 4011, 4019, 40501, 40509, 40511, 40519, 40591, 40599, I10, I15, I150, I151, I152, I158, I159, O16, O161, O162, O163, O169 |
| Diabetes (DM) | 24900, 24901, 24910, 24911, 24920, 24921, 24930, 24931, 24940, 24941, 24950, 24951, 24960, 24961, 24970, 24971, 24980, 24981, 24990, 24991, 25000, 25001, 25002, 25003, 25010, 25011, 25012, 25013, 25020, 25021, 25022, 25023, 25030, 25031, 25032, 25033, 25040, 25041, 25042, 25043, 25050, 25051, 25052, 25053, 25060, 25061, 25062, 25063, 25070, 25071, 25072, 25073, 25080, 25081, 25082, 25083, 25090, 25091, 25092, 25093, E08, E080, E0800, E0801, E081, E0810, E0811, E082, E0821, E0822, E0829, E083, E0831, E08311, E08319, E0832, E08321, E083211, E101, E083212, E083213, E083219, E08329, E083291, E083292, E083293, E083299, E0833, E08331, E083311, E083312, E083313, E083319, E08339, E083391, E083392, E083393, E083399, E0834, E08341, E083411, E083412, E083413, E083419, E08349, E083491, E083492, E083493, E083499, E0835, E08351, E083511, E083512, E083513, E083519, E08352, E083521, E083522, E083523, E083529, E08353, E083531, E083532, E083533, E083539, E08354, E083541, E083542, E083543, E083549, E08355, E083551, E083552, E083553, E083559, E08359, E083591, E083592, E083593, E083599, E0836, E0837, E0837X1, E0837X2, E0837X3, E0837X9, E0839, E084, E0840, E0841, E0842, E0843, E0844, E0849, E085, E0851, E0852, E0859, E086, E0861, E08610, E08618, E0862, E08620, E08621, E08622, E08628, E0863, E08630, E08638, E0864, E08641, E08649, E0865, E0869, E088, E089, E09, E090, E0900, E0901, E091, E0910, E0911, E092, E0921, E0922, E0929, E093, E0931, E09311, E09319, E0932, E09321, E093211, E093212, E093213, E093219, E09329, E093291, E093292, E093293, E093299, E0933, E09331, E093311, E093312, E093313, E093319, E09339, E093391, E093392, E093393, E093399, E0934, E09341, E093411, E093412, E093413, E093419, E09349, E093491, E093492, E093493, E093499, E0935, E09351, E093511, E093512, E093513, E093519, E09352, E093521, E093522, E093523, E093529, E09353, E093531, E093532, E093533, E093539, E09354, E093541, E093542, E093543, E093549, E09355, E093551, E093552, E093553, E093559, E09359, E093591, E093592, E093593, E093599, E0936, E0937, E0937X1, E0937X2, E0937X3, E0937X9, E0939, E094, E0940, E0941, E0942, E0943, E0944, E0949, E095, E0951, E0952, E0959, E096, E0961, E09610, E09618, E0962, E09620, E09621, E09622, E09628, E0963, E09630, E09638, E0964, E09641, E09649, E0965, E0969, E098, E099, E10, E1010, E1011, E102, E1021, E1022, E1029, E103, E1031, E10311, E10319, E1032, E10321, E103211, E103212, E103213, E103219, E10329, E103291, E103292, E103293, E103299, E1033, E10331, E103311, E103312, E103313, E103319, E10339, E103391, E103392, E103393, E103399, E1034, E10341, E103411, E103412, E103413, E103419, E10349, E103491, E103492, E103493, E103499, E1035, E10351, E103511, E103512, E103513, E103519, E10352, E103521, E103522, E103523, E103529, E10353, E103531, E103532, E103533, E103539, E10354, E103541, E103542, E103543, E103549, E10355, E103551, E103552, E103553, E103559, E10359, E103591, E103592, E103593, E103599, E1036, E1037, E1037X1, E1037X2, E1037X3, E1037X9, E1039, E104, E1040, E1041, E1042, E1043, E1044, E1049, E105, E1051, E1052, E1059, E106, E1061, E10610, E10618, E1062, E10620, E10621, E10622, E10628, E1063, E10630, E10638, E1064, E10641, E10649, E1065, E1069, E108, E109, E11, E110, E1100, E1101, E111, E1110, E1111, E112, E1121, E1122, E1129, E113, E1131, E11311, E11319, E1132, E11321, E113211, E113212, E113213, E113219, E11329, E113291, E113292, E113293, E113299, E1133, E11331, E113311, E113312, E113313, E113319, E11339, E113391, E113392, E113393, E113399, E1134, E11341, E113411, E113412, E113413, E113419, E11349, E113491, E113492, E113493, E113499, E1135, E11351, E113511, E113512, E113513, E113519, E11352, E113521, E113522, E113523, E113529, E11353, E113531, E113532, E113533, E113539, E11354, E113541, E113542, E113543, E113549, E11355, E113551, E113552, E113553, E113559, E11359, E113591, E113592, E113593, E113599, E1136, E1137, E1137X1, E1137X2, E1137X3, E1137X9, E1139, E114, E1140, E1141, E1142, E1143, E1144, E1149, E115, E1151, E1152, E1159, E116, E1161, E11610, E11618, E1162, E11620, E11621, E11622, E11628, E1163, E11630, E11638, E1164, E11641, E11649, E1165, E1169, E118, E119, E13, E130, E1300, E1301, E131, E1310, E1311, E132, E1321, E1322, E1329, E133, E1331, E13311, E13319, E1332, E13321, E133211, E133212, E133213, E133219, E13329, E133291, E133292, E133293, E133299, E1333, E13331, E133311, E133312, E133313, E133319, E13339, E133391, E133392, E133393, E133399, E1334, E13341, E133411, E133412, E133413, E133419, E13349, E133491, E133492, E133493, E133499, E1335, E13351, E133511, E133512, E133513, E133519, E13352, E133521, E133522, E133523, E133529, E13353, E133531, E133532, E133533, E133539, E13354, E133541, E133542, E133543, E133549, E13355, E133551, E133552, E133553, E133559, E13359, E133591, E133592, E133593, E133599, E1336, E1337, E1337X1, E1337X2, E1337X3, E1337X9, E1339, E134, E1340, E1341, E1342, E1343, E1344, E1349, E135, E1351, E1352, E1359, E136, E1361, E13610, E13618, E1362, E13620, E13621, E13622, E13628, E1363, E13630, E13638, E1364, E13641, E13649, E1365, E1369, E138, E139 |
| Coronary heart disease (CHD) | 41400, 41401, 41402, 41403, 41404, 41405, 41406, 41407, 4143, 4144, I2101, I2102, I2109, I2111, I2119, I2121, I240, I251, I2510, I2511, I25110, I25111, I25118, I25119, I257, I2570, I25700, I25701, I25708, I25709, I2571, I25710, I25711, I25718, I25719, I2572, I25720, I25721, I25728, I25729, I2573, I25730, I25731, I25738, I25739, I2575, I25750, I25751, I25758, I25759, I2576, I25760, I25761, I25768, I25769, I2579, I25790, I25791, I25798, I25799, I2581, I25810, I25811, I25812, I2582, I2583, I2584, T822, T8221, T82211, T82211A, T82211D, T82211S, T82212, T82212A, T82212D, T82212S, T82213, T82213A, T82213D, T82213S, T82218, T82218A, T82218D, T82218S, T82855, T82855A, T82855D, T82855S, Z951, Z955, Z9861 |
| Acute or chronic heart failure (HF) | 39891, 40201, 40211, 40291, 40401, 40403, 40411, 40413, 40491, 40493, 4280, 4281, 42820, 42821, 42822, 42823, 42830, 42831, 42832, 42833, 42840, 42841, 42842, 42843, 4289, I0981, I110, I130, I132, I50, I502, I5020, I5021, I5022, I5023, I503, I5030, I5031, I5032, I5033, I504, I5040, I5041, I5042, I5043, I508, I5081, I50810, I50811, I50812, I50813, I50814, I5082, I5083, I5084, I5089, I509, I9713, I97130, I97131 |
| Chronic kidney disease (CKD) | 28521, 40300, 40301, 40310, 40311, 40390, 40391, 40400, 40401, 40402, 40403, 40410, 40411, 40412, 40413, 40490, 40491, 40492, 40493, 5851, 5852, 5853, 5854, 5855, 5859, D631, E0822, E0922, E1022, E1122, E1322, I12, I120, I129, I13, I130, I131, I1310, I1311, I132, N18, N181, N182, N183, N184, N185, N189 |
| Liver disease | According to the Charlson comorbidity index materialized view |
| Chronic pulmonary disease | According to the Charlson comorbidity index materialized view |
| Malignant cancer | According to the Charlson comorbidity index materialized view |

**Table S2** The list of oral antihypertensive drugs included in present study

|  | Antihypertensive drugs |
| --- | --- |
| ACEIs | Benazepril, Captopril, Enalapril, Fosinopril, Lisinopril, Moexipril, Monopril, Perindopril, Quinapril, Ramipril, Trandolapril |
| ARBs | Candesartan, Irbesartan, losartan, Olmesartan, Telmisartan, Valsartan |
| β-Blockers | Acebutolol, Atenolol, Betaxolol, Bisoprolol, Labetalol, Metoprolol, Nadolol, Nebivolol, Penbutolol, Pindolol, Propranolol |
| Dihydropyridine CCBs | Amlodipine, Felodipine, Isradipine, Nifedipine, Nimodipine, Nisoldipine, |
| Non-dihydropyridine CCBs | Diltiazem, Verapamil |
| Thiazide diuretics | Chlorothiazide, Hydrochlorothiazide, Metolazone |


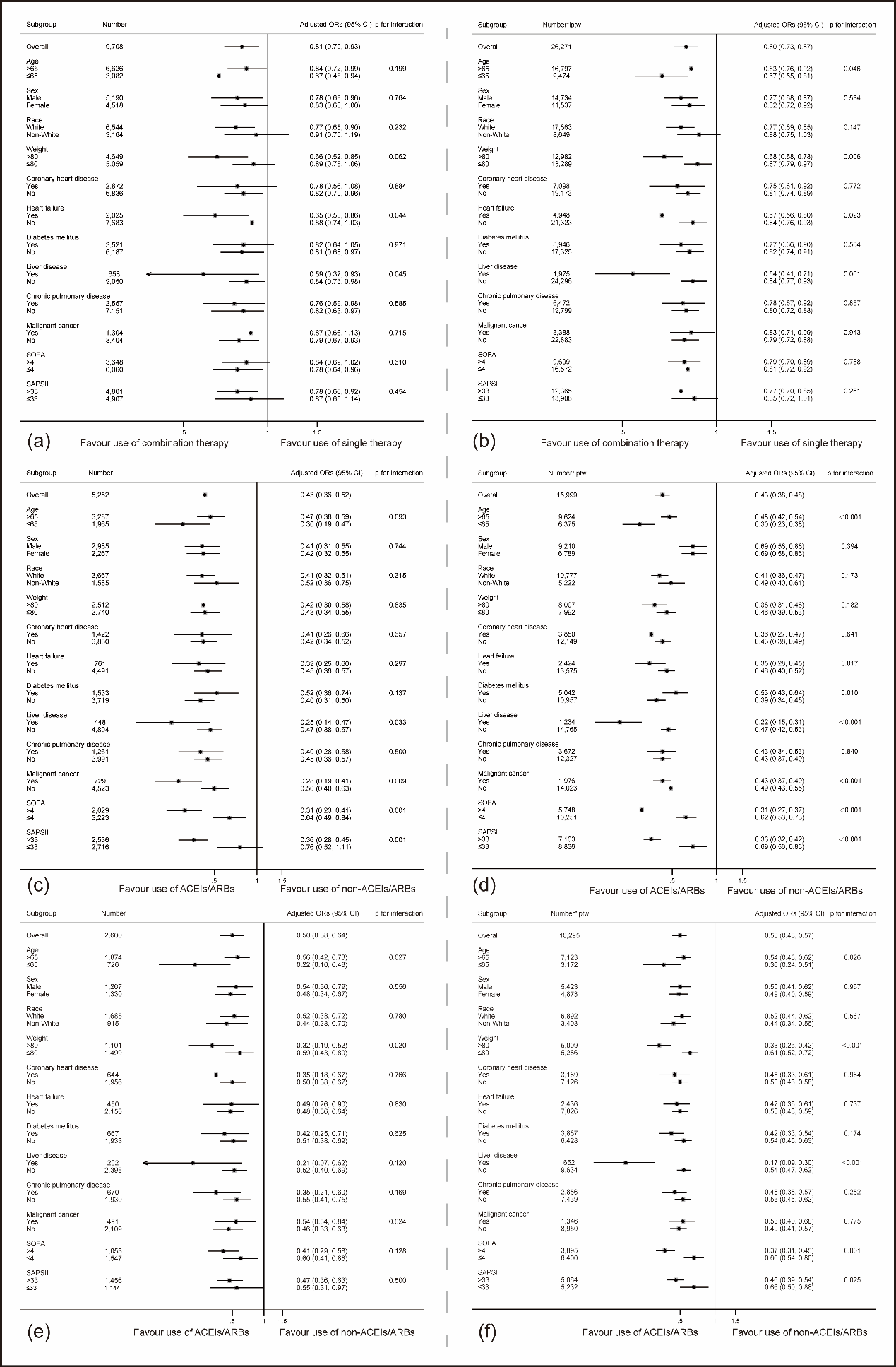


**Figure S1** The result of subgroup analysis in PSM and IPTW cohorts. (a,b) show the results of subgroup analyses in the comparing combined and single therapy. (c,d) show the results of subgroup analyses regarding the protective effect of ACEIs/ARBs exposure in the single antihypertensive drug exposure cohort, and (e,f) show the protective effect of ACEIs/ARBs in the combined therapy cohort. (a,c,e) represent results from the PSM cohorts, while (b,d,f) represent results from the IPTW cohorts
